# Sequencing and analysis of the complete mitochondrial genome of the lesser bandicoot rat (*Bandicota bengalensis*) from China and its phylogenetic analysis

**DOI:** 10.1080/23802359.2021.1942273

**Published:** 2021-06-21

**Authors:** Liu Zhu, Wang Qing-Qing, Jiang Xue-Ting, Jiang Wen-Jing, Zhao Xin-Xu, Yao Qian-Qian, Zhang Jun-Sheng, Piao Zhong-Wan

**Affiliations:** College of Life Science and Technology, Mudanjiang Normal University, Mudanjiang, P.R. China

**Keywords:** Control region, mitogenome, phylogenetic trees, *Bandicota bengalensis*

## Abstract

The complete mitogenome sequence of the lesser bandicoot rat (*Bandicota bengalensis* Gray and Hardwicke, 1833) was determined using long PCR. The genome was 16,327 bp in length and contained 13 protein-coding genes, 2 ribosomal RNA genes, 22 transfer RNA genes, 1 origin of L strand replication and 1 control region. The overall base composition of the heavy strand is A (34.2%), C (24.9%), T (28.5%) and G (12.4%). The base compositions present clearly the A–T skew, which is most obviously in the control region and protein-coding genes. Mitochondrial genome analyses based on MP, ML, NJ and Bayesian analyses yielded identical phylogenetic trees. This study verifies the evolutionary status of *Bandicota bengalensis* in Muridae at the molecular level. The mitochondrial genome would be a significant supplement for the *Bandicota bengalensis* genetic background. The two *Bandicota* species formed a monophyletic group with the high bootstrap value (100%) in all examinations.

The lesser bandicoot rat (*Bandicota bengalensis* Gray and Hardwicke, 1833) is a murid rodent distributed mostly in Asia that can cause substantial negative economic impact in urban and rural areas. In this paper, A muscle sample was obtained from a female *Bandicota bengalensis* captured from Ruili regions in Yunnan Province, China (24°01′12″ N, 97°85′18″ E). The muscle tissue was preserved in 95% ethanol and stored at −75 °C before use. The specimen and its DNA is stored in Animal and Plant Herbarium of Mudanjiang Normal University. The voucher number is XBCS2019004 (liuzhu, swxlz0@126.com). Genomic DNA was extracted from muscle using the EasyPure genomic DNA kit (TransGen Biotech Co., Beijing, China). The mitogenomes were sequencing by Illumina NovaSeq 6000 platform (Ruiboxingke Biotechnology Co. Ltd., Beijing, China) using a primer walking strategy and the long and accurate PCR. The draft sequence was manually corrected. The complete mitochondrial genome sequence was annotated using Sequin.

The mitochondrial genome is a circular double-stranded DNA sequence that is 16,327 bp long including 13 protein-coding genes, 2 rRNA genes, 22 tRNA genes, 1 origin of L strand replication and 1 control region. The accurate annotated mitochondrial genome sequence was submitted to GenBank with accession number MW364625. The arrangement of the multiple genes is in line with other Muridae species (Robins et al. 2008; Chen et al. 2012; Jing et al. 2015; Chang et al. 2016; Yong et al. 2016; Zhang et al. 2016; Wei et al. 2017; Lv et al. 2019) and most mammals (Mouchaty et al. 2000; Nikaido et al. 2001; Nikaido et al. 2003; Fontanillas et al. 2005; Cabria et al. 2006; Meganathan et al. 2012; Yoon et al. 2013; Xu et al. 2012, 2013; Kim et al. 2013, 2017; Hou et al. 2016; Huang et al. 2014, 2016; Xu et al. 2016; Liu et al. 2016; Liu, Tian, Jin, Jin, et al. 2017; Liu, Tian, Jin, Dong, et al. 2017; Liu, Wang, et al. 2017; Liu et al. 2018; Liu, Dang, et al. 2019; Liu, Qin, et al. 2019; Jin et al. 2017; Gutiérrez et al. 2018; Jia et al. 2018).

The control region of *Bandicota bengalensis* mitochondrial genome was located between the tRNA-Pro and tRNA-Phe genes, and contains only promoters and regulatory sequences for replication and transcription, but no structural genes. Three domains were defined in *Bandicota bengalensis* mitochondrial genome control region (Zhang et al. 2009): the extended termination-associated sequence (ETAS) domain, the central conserved domain (CD) and the conserved sequence block (CSB) domain.

The total length of the protein-coding gene sequences was 11,405 bp. Most protein-coding genes initiate with ATG except for ND1, ND3 and ND5, which began with GTG or ATT. Eleven protein-coding genes terminated with TAA. The incomplete stop codons (T– –) were used in COX3, ND4 and Cyt b. A strong bias against A at the third codon position was observed in the protein-coding genes. The frequencies of CTA (Leu), ATT (Ile), TTA (Leu) and ATA (Met) were higher than those of other codons. The length of tRNA genes varied from 58 to 77 bp.

Most *Bandicota bengalensis* mitochondrial genes were encoded on the H strand, except for the ND6 gene and eight tRNA genes, which were encoded on the L strand. Some reading frame intervals and overlaps were found. One of the most typical was between ATP8 and ATP6. The L-strand replication origin (OL) was 32 bp long and had the potential to fold into a stable stem-loop secondary structure. The total base composition of *Bandicota bengalensis* mitochondrial genome was A (34.2%), C (24.9%), T (28.5%) and G (12.4%). The base compositions clearly present the A-T skew, which was most obviously in the control region and protein coding genes.

In order to explore the evolution of Muridae species, especially the evolution of genus *Bandicota* from China, here, we investigate the molecular phylogenetics of Chinese *Bandicota bengalensis* using complete mitochondrial genome sequence of 36 species. All sequences generated in this study have been deposited in the GenBank (Figure 1).

**Figure 1.**
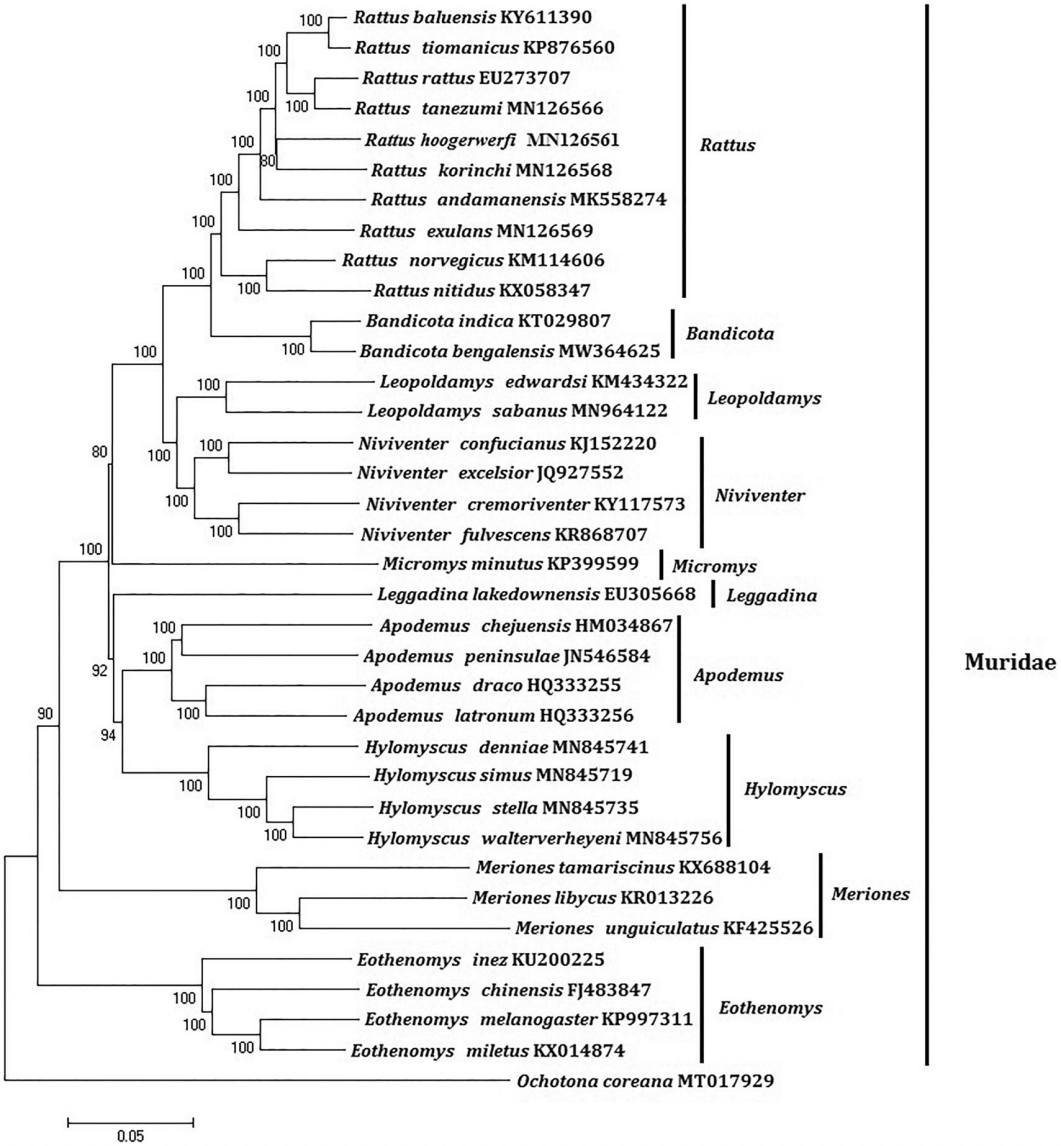
Phylogenetic tree generated using the Maximum Parsimony method based on complete mitochondrial genomes. The out group is *Ochotona coreana* (MT017929).

Mitochondrial genome analyses based on MP, ML, NJ and Bayesian analyses yielded identical phylogenetic trees, indicating a close phylogenetic affinity of species. The phylogram obtained from ML method is shown in Figure 1. It shows that one major phyletic lineages were present in Muridae. In this study, the 10 genera (*Rattus*, *Niviventer*, *Bandicota*, *Hylomyscus*, *Leopoldamys*, *Apodemus*, *Micromys*, *Eothenomys*, *Leggadina*, and *Meriones*) included in Muridae form independent branches. *Bandicota* comprised *Bandicota bengalensis* and *Bandicota indica* was supported by bootstrap values of 100%. This study verifies the evolutionary status of *Bandicota bengalensis* in Muridae at the molecular level. The mitochondrial genome would be a significant supplement for the *Bandicota bengalensis* genetic background. The two *Bandicota* species formed a monophyletic group with the high bootstrap value (100%) in all examinations.

## Data Availability

The data that support the findings of this study are openly available in GenBank at https://www.ncbi.nlm.nih.gov/, reference number MW364625.
